# Distinctive patterns of epigenetic marks are associated with promoter regions of mouse LINE-1 and LTR retrotransposons

**DOI:** 10.1186/1759-8753-4-27

**Published:** 2013-12-02

**Authors:** Danny Rangasamy

**Affiliations:** 1 John Curtin School of Medical Research, The Australian National University, Canberra, ACT 2601, Australia

**Keywords:** LINE-1, LTR, Chromatin, Histone, Heterochromatin, Transcriptional silencing

## Abstract

**Background:**

The long terminal repeat (LTR) retrotransposons and the non-LTR retrotransposons (LINE-1 or L1) make up more than one-third of the mouse genome. Because of their abundance, the retrotransposons are the major players in genomic structure and function. While much attention has been focused on the biology of retrotransposons, little is known about the chromatin structure of these elements or the potential role of epigenetic marks on the regulation of retrotransposon expression.

**Findings:**

Using sequential chromatin immunoprecipitation analysis, we analyzed the cohabitation of several post-translational histone modifications in the promoter regions of mouse L1 and LTR retrotransposons. We show here that the variant histone H2A.Z selectively present in L1 promoters. Notably, H2A.Z and trimethylated histone H3 (H3K9me3) co-localize in the same genomic location of the L1 promoter along with heterochromatin-binding protein HP1α. In contrast, *Mm*ERV and intracisternal A-particle (IAP) classes of LTR promoters are enriched with core histone H2A and heterochromatic trimethylated histone H4 (H4K20me3). These distinctive patterns of chromatin modifications are relatively consistent irrespective of cell type.

**Conclusions:**

Chromatin structure regulates the expression of retrotransposons. LINE-1 elements are associated with H2A.Z and HP1α-containing constitutive heterochromatin, while the LTR elements are enriched with H2A and the H4K20me3-type of facultative heterochromatin. Our findings demonstrate that different epigenetic mechanisms operate in the mouse genome to silence different classes of retrotransposons.

## Findings

### Introduction

In the nucleus, DNA is packaged into a protein complex known as chromatin. Roughly 150 bp of DNA are wrapped around a histone octomer, consisting of two copies of ‘core’ histones (H2A, H2B, H3 and H4), to form a basic unit of chromatin. Histone proteins form the majority of chromatin and their post-translational modifications of amino-terminal tails (PTMs) affect most of the processes that occur in DNA including gene expression, replication and mitosis [1]. Many histone-modifying enzymes regulate histone PTM states, which occur mostly at the level of lysine, namely lysine acetylation, mono- (me1), di- (me2) and trimethylation (me3). Acetylation is essentially correlated with gene activation, being localized to transcriptional start sites of potentially active genes. In contrast, the correlation of methylation with gene expression depends on its level and the residue. Methylation on histone H3 lysine K4 and K9 is associated with activation and repression of genes, respectively. Another feature associated with PTM is the substitution of core histones with the corresponding histone variants [2,3]. The majority of PTMs recruit transcriptional factors and other non-histone proteins to chromatin that mediate many events including transcriptional activation or silencing of genes. Recently, it has become clear that the structure of chromatin plays an important role in the regulation of retrotransposons [4,5]. While most are inactive, some retrotransposons are still potentially active in the genome. In order to inactivate those harmful elements, cells silence their expression via DNA methylation and packaging into chromatin associated with repressive histone marks. In humans, DNA methylation at LINE-1 or L1 promoters has been associated with transcriptional control of L1 elements [6]. In addition, L1 elements are also subjected to histone H3 trimethylation (H3K9me3), histone deacetylation (HDACs), and in some cases the RNAi machinery guides the epigenetic control of L1 expression [7,8]. These chromatin modifications are functionally interrelated and coordinate epigenetic silencing of retrotransposons. However, the chromatin states responsible for transcriptional control of retrotransposons in mouse somatic cells remain poorly understood. To explore these interrelationships, we used the conventional chromatin immunoprecipitation (ChIP) assay to analyze the global distribution of individual histone modifications (H2A, H2A.Z and AcH2A.Z) as well as sequential chromatin immunoprecipitation (SeqChIP) assays to analyze the cohabitation of multiple histone modifications (H3K4me2, H3K9me3, AcH4K16, H4K20me3, HP1α) at the same genomic locations of L1 and LTR promoters in two most commonly used mouse cell lines, NIH3T3 and L929. We found that the promoters of L1 and LTR retrotransposons possess distinct patterns of repressive histone marks irrespective of mouse cell type.

## Methods

Mouse fibroblast cells (NIH3T3 and L929) were obtained from ATCC and maintained in Dulbecco’s modified Eagle’s medium (DMEM) with 2 mM L-glutamine and 10% FCS at 37°C under 5% CO_2_. A conventional ChIP assay was carried out as described in the protocol for the Active Motif’s Magnetic ChIP-IT Express Kit (Active Motif, Carlsbad, CA, USA). Briefly, 100 μg of chromatin fragments were immunoprecipitated with 15 μg of H2A or H2A.Z antibodies [9], or with 15 μg of AcH2A.Z antibodies (Abcam#ab18262). DNA from the antibody-bound fractions was purified by Proteinase K and phenol/chloroform extractions. DNA was ethanol precipitated and subjected to real-time qPCR analysis. Percent enrichment was calculated by 100 x 2^^^(ΔC_Tadjusted input_ - ΔC_Tenriched_). Input DNA ΔC_T_ value was adjusted from 1% to 100% equivalent to substrating 6.64 ΔC_T_s or log_2_ 100. As a positive control, the enriched DNA was also probed with specific primers for GAPDH promoter DNA in qPCR analysis [see Additional file 1: Figure S1].

The sequential ChIP assays were performed as described in the Re-ChIP protocols from Active Motif with the exception that a protein-protein crosslinking step with dimethyl 3,3’-dithiobispropionimidate (DTBP) was added prior to formaldehyde fixation [9]. The first ChIP was performed with 600 μg of input chromatin with 120 μg of H2A or H2A.Z antibodies and the eluted chromatin was divided equally into six aliquots. Samples not used for second ChIP were decrosslinked and purified by Proteinase K and phenol/chloroform extractions. For SeqChIP, each aliquot of chromatin was subsequently used in the second ChIP reaction with 20 μg of H3K4me2 (abcam#32356), H3K9me3 (activemotif#39161), AcH4K16 (abcam#46983), HP1α (millipore#MAB3446), H4K20me3 (millipore#07-463) and H2A (abcam#18255) or H2A.Z (millipore#17-10048) antibodies. Aliquots of input DNA before and after performing the first ChIP reaction were used as controls in qPCR analysis together with the second ChIP samples. As a negative control, mouse IgG was used for ‘no antibody’ or non-specific ChIP. DNA from input and from first and second ChIP reactions was quantified using PicoGreen® dsDNA dye (Invitrogen) before subjecting it to real-time qPCR analysis, followed by 2% agarose gel electrophoresis. Real-time PCR was performed in triplicate using 5 to 10 ng ChIP DNA or Input DNA in a 25 μl reaction containing 1X SYBR Green Supermix (Bio-Rad) and 400 nM each of forward and reverse primers. Relative enrichment values were calculated by normalizing the ΔC_T_ value of second ChIP DNA to the ΔC_T_ of first ChIP DNA input with substation of the mouse IgG antibody background. Co-occupancy of the SeqChIP was calculated from the percent of first ChIP-DNA that also contains second ChIP DNA.

The oligonucleotide primers used are as follows: LINE-1 5’-UTR promoter (monomers) forward 5’-AGC TTC TGG AAC AGG CAG AA-3’; reverse 5’-CAC TGT GTT GCT TTG GCA GT-3’; LINE-1 non-monomer forward 5’-GAG GAG GCC CAA ATA CAA GA-3’; reverse 5’-TGT TTA GAG ATT GTT CTT CTG GTG A-3’; SINE B1 forward 5’-GGT GTG GTG GCG CAC ACC-3’; reverse 5’-CCT GGC TGT CCT GGA GCT C-3’; IAP forward 5’-AAG GGA CGG GGT TTC GTT TT-3’; reverse 5’-ACT GGT ACT CTC GTT CCC CA-3’; *Mm*ERV forward 5’-AAC TCG TTC CCA GAA CACT CC-3’; reverse 5’- AGC GGG GTA GGG AAA GTA CAA-3’; GADPH forward 5’-TAC TAG CGG TTT TAC GGG CG-3’; reverse 5’-TCG AAC AGG AGG AGC AGA GAG CGA-3’. *In silico* analysis of UCSC Primer-Blast (GRCm38/mm10) suggests that the primers used for mouse L1 promoters (product size 154 bp and 230 bp for monomers and non-monomer region, respectively) can detect the following LINE-1 subfamilies: L1Md_T, L1Md_GF and L1Md_A. The IAP primers (product size 310 bp) amplify the IAPLTR1a_*M*m subfamily, and *Mm*ERV primers amplify (product size 260 bp) the RLTR6-*M*m subfamily.

## Results

**Figure 1:**
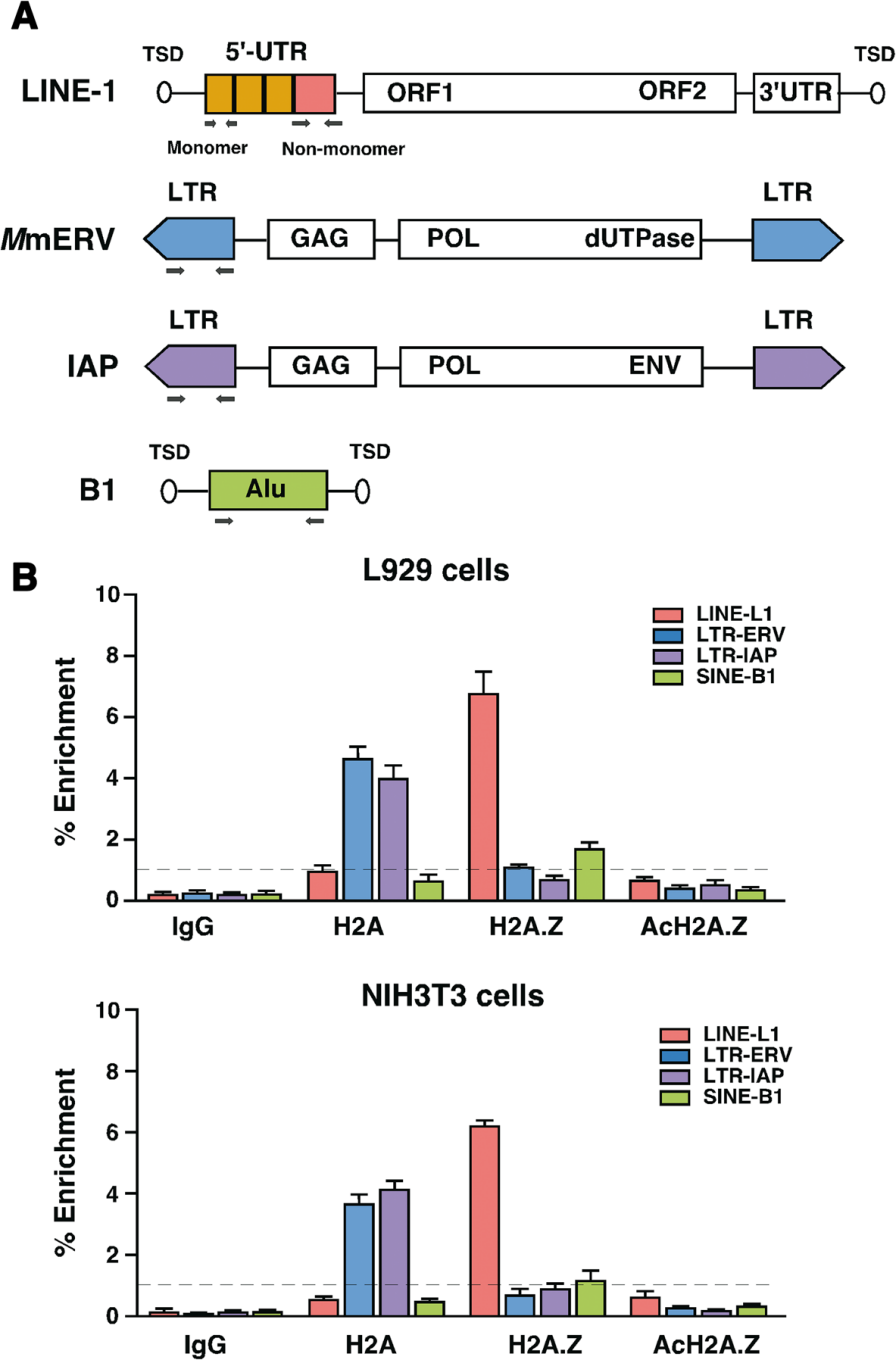
**H2A and H2A.Z are selectively enriched with long terminal repeat (LTR) and long interspersed nuclear element-1 (LINE-1) elements, respectively. (A)** Schematic representations of full-length LINE-1, LTR (*M*mERV and IAP) and SINE-B1 elements. The position of the primer binding sites is indicated in arrows. **(B)** Chromatin immunoprecipitation (ChIP) was performed with anti-H2A, anti-H2A.Z or anti-AcH2A.Z affinity purified antibodies on chromatin preparations harvested from L929 and NIH3T3 cells. IgG represents non-specific background after immunoprecipitation. Genomic DNA was extracted and quantified by qPCR using consensus primers specific for the three classes of L1, LTR (intracisternal A-particle (IAP) and mouse endogenous retrovirus (*Mm*ERV)) and B1 retrotransposons. Enrichment was determined by 100 x 2^^^ (ΔCT_input_ - ΔCT_enriched_) after adjusting the percentage of input DNA used. Shown are average ± s.d. of three biological replicates (n).

Cells normally deploy a number of genomic defense mechanisms to guard against the harmful consequences of retrotransposon activity. Retrotransposons are rarely expressed in normal cells, thus we hypothesized that chromatin status could exert considerable influence on the functional state of retrotransposons. To explore whether different retrotransposons possess specific chromatin modifications, we isolated chromatin fragments (ranging from 200 bp to 600 bp containing 2 to 4 nucleosome arrays [see Additional file 1: Figure S1a]) from mouse cell lines and performed conventional ChIP assays with the major (core) histone H2A and its corresponding histone variant H2A.Z to survey nucleosome occupancy. The negative and positive controls were, respectively, non-specific precipitation with mouse IgG antibody or probed against the GAPDH promoter [see Additional file 1: Figure S1b]. Genomic DNA associated with H2A, and H2A.Z chromatin-bound fragments was purified, and using consensus primers to detect multiple copies of L1, SINE-B1, and LTR (*Mm*ERV and IAP)-elements, we subjected it to qPCR analysis to determine the relative abundance of H2A and H2A.Z in different retrotransposons (Figure 1A). The results show that the relative enrichment of H2A.Z at the promoter regions of L1 was at least six fold higher than that of H2A (Figure 1B). Notably, the primers used for L1 PCR are expected to bind and amplify multiple copies of mouse LINE-1 promoters, including recently evolved L1Md_T subfamily, together with the L1Md_GF and L1Md_A subfamilies. In contrast, H2A was enriched across the promoters of the *Mm*ERV (ERV1 family) and IAP (ERVK family) class of LTR elements. In the case of SINE-B1 (Alu family) elements, we did not find any significant enrichment of H2A or H2A.Z, although the relative enrichment of H2A.Z was slightly higher than that seen for H2A. This observation suggests that SINE elements might contain different forms of H2A variants such as macroH2A1, macroH2A2 or other PTMs of H2A. The two mouse cells used in our study (L929 and NIH3T3) exhibited similar histone profiles across the promoter regions of retrotransposons. It has previously been reported that H2A.Z can be acetylated at lysines 4, 7 and 11 and the presence or the absence of acetylated H2A.Z (acH2A.Z) is associated with transcriptional activation or silencing of genes, respectively [10]. We therefore tested whether H2A.Z is present in its acetylated forms. To do this, we repeated the ChIP assay using an antibody specific for acH2A.Z and found that, unlike H2A.Z enrichment, there was a clear deficiency of acH2A.Z across the promoters of L1 and LTR elements (Figure 1B panel AcH2A.Z), suggesting that H2A.Z is in its deacetylated isoform and is associated with silencing state of genes. These qualitative findings raised the intriguing possibility that different histone modifications might be associated with different classes of mouse retrotransposons.

**Figure 2:**
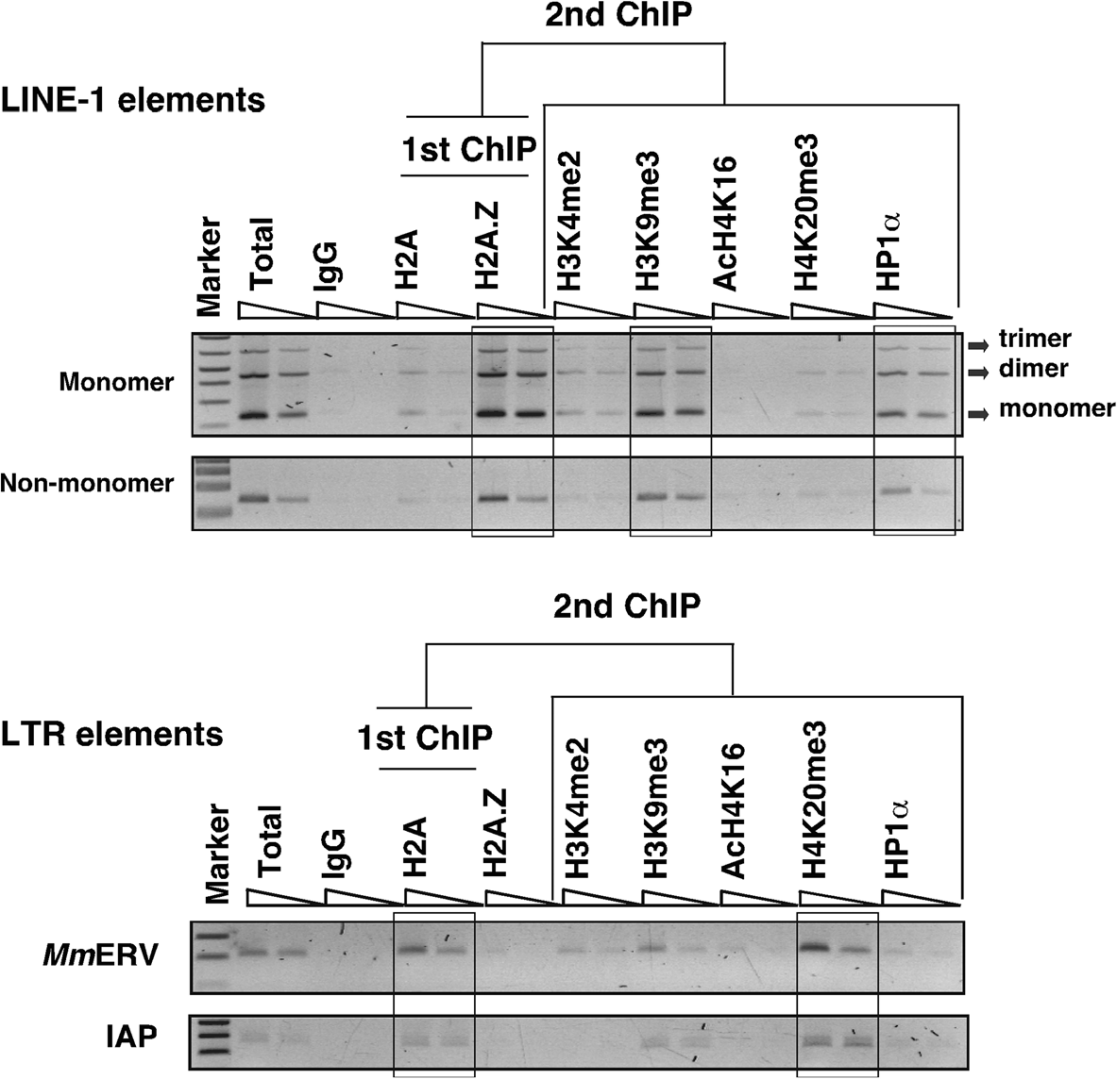
**Selective enrichment of repressive histone marks at the long interspersed nuclear element-1 (L1) and long terminal repeat (LTR) promoters.** Shown is representative sequential chromatin immunoprecipitation (SeqChIP) raw data using the first chromatin immunoprecipitation (ChIP) to H2A or H2A.Z antibodies followed by the second ChIP to H3K4me2, H3K9me3, AcH4K16, H4K20me3 and HP1α antibodies. About 1% of the sonicated chromatin was set aside as total input DNA before the ChIP assays. DNA was purified following crosslink reversal and used as positive controls. Mouse IgG ChIP served as negative controls. Dilutions of 10 and 5 ng of ChIP DNA were used to determine the relative abundance of histone marks at the promoter regions of L1 and LTR retrotransposons. One-tenth of each PCR product was visualized on a 3% agarose gel. Marker, 1 kb-plus DNA marker. Top panel shows the relative enrichment of H2A.Z, H3K9me3 and HP1α as unison at the L1 promoter that includes tandem repeats of L1 monomers whereas the bottom panel shows the enrichment of H2A in the promoters of MmERV and IAP classes of LTR elements.

**Figure 3:**
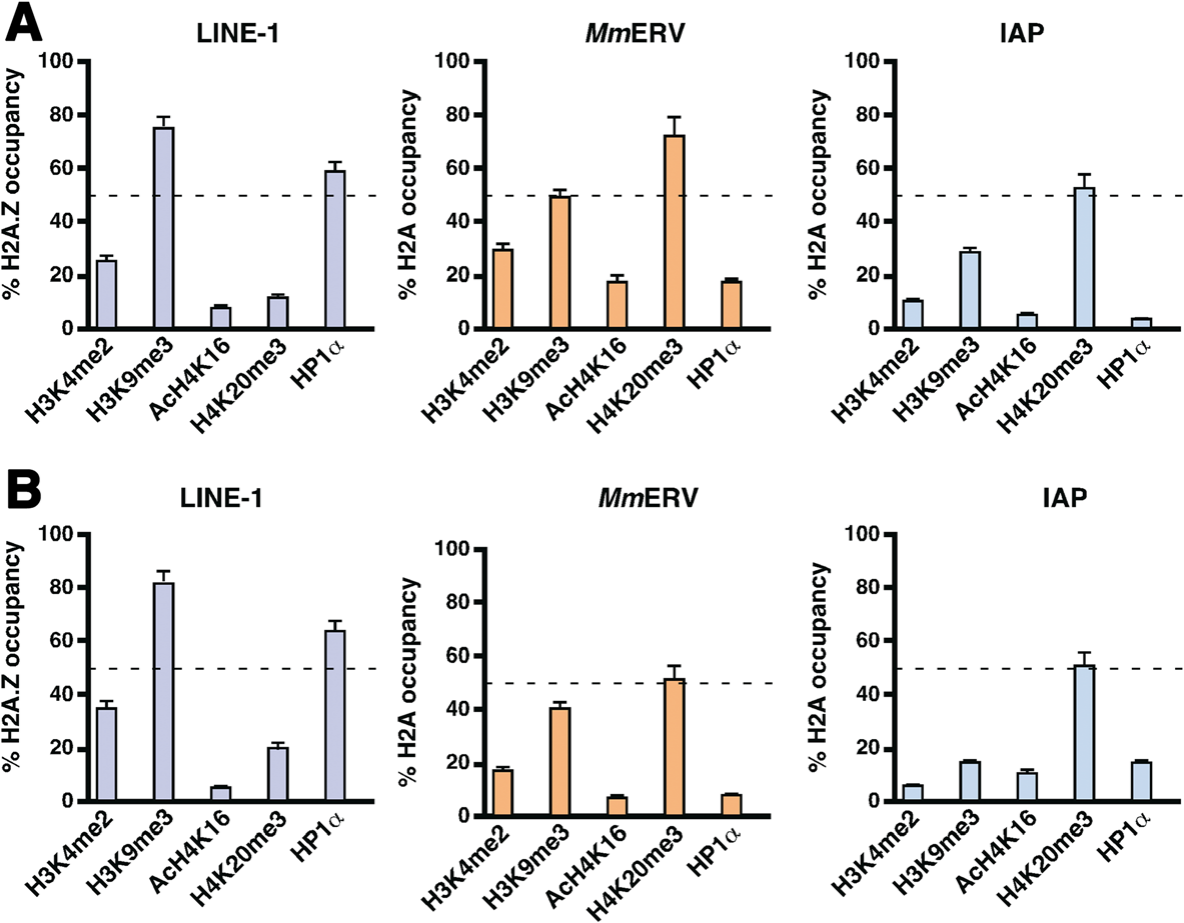
**Co**-**occupancy of histone marks at the interspersed nuclear element-1 (L1) and long terminal repeat (LTR) promoters.** Sequential chromatin immunoprecipitation (SeqChIP) was performed on chromatin from L929 **(A)** and NIH3T3 **(B)** cells using H2A and H2A.Z antibodies, followed by the second ChIP reactions as described above. The resulting enriched DNA was probed for LINE-1, LTR (mouse endogenous retrovirus (*Mm*ERV) and intracisternal A-particle (IAP)), and SINE-B1 using consensus primers by qPCR. The relative enrichment was calculated as the percentage of the first ChIP for H2A or H2A.Z-containing DNA that also contains second ChIP DNA. Percent occupancy above 50% was considered as significant. Error bars, s.d. of three biological replicates.

H2A.Z occupancy often influences other histone components within the nucleosome that can modulate different chromatin structures [9]. With the aim of deciphering the combinatorial influence of histones, we determined whether other histone modifications simultaneously co-occupy with the same region of retrotransposon DNA. To do this, we performed sequential ChIP (SeqChIP) assays (also known as ‘Re-ChIP’ assays) which involve two consecutive immunoprecipitations [11]. The first ChIP was performed with H2A or H2A.Z antibodies like conventional ChIP. Genomic DNA associated with H2A, and H2A.Z chromatin-bound fragments was purified and subjected to the second ChIP with H3K4me2, H3K9me3, AcH4K16, H4K20me3 and HP1α antibodies to determine possible co-occupancy with H2A and H2A.Z immunoprecipitated proteins. In this analysis, we used the arbitrary cutoff of 50%. The use of a cutoff value removes weaker signals of ChIP and highlights only the stronger signals [11]. Remarkably, we found that the majority of H2A.Z, H3K9me3 and HP1α binding co-localizes across the entire 5’-UTR sequence of the L1 promoters that include tandem repeats of approximately 200-bp monomers and non-monomeric region of the L1 promoter (Figure 2, top panel). Co-occupancy analysis of the SeqChIP assay revealed that H2A.Z, H3K9me3 and HP1α are always present in unison at the L1 promoter but not with active H3K4me2 marker or repressive H4K20me3 marker (Figure 3). These observations are consistent with our earlier studies that H2A.Z can interact with constitutive heterochromatin-binding protein, HP1α, to reinforce the silenced state of genes [9].

To investigate whether LTR elements are also associated with similar repressive markers, we generated SeqChIP profiles for H2A in the same genomic DNA of cells as above. As expected, we found that H2A was enriched at LTRs and that the relative abundance of H2A was similar in the promoters of the *Mm*ERV and IAP class of LTR elements (Figure 2, bottom panel). This enrichment was not cell type-dependent given that both L929 and NIH3T3 cells produced similar profiles (Figure 3). Importantly, our SeqChIP analysis showed that, unlike the LINE-L1 promoter, there was no significant enrichment of HP1α. Instead, H2A was co-localized with trimethylated histone H4 (H4K20me3), a well-known marker for highly compacted facultative heterochromatin regions [12,13]. Although a fraction of H3K9me3 marker appear to be enriched with H4K20me3 at the LTR promoters, similar to mouse embryonic cells [5], the relative enrichment of H4K20me3 was greater than that of H3K9me3 in both L929 and NIH3T3 cells (Figure 3). Strikingly, we found no enrichment of the active chromatin marker (AcH4K16) across the promoter regions, indicating that LTRs are indeed associated with the H4K20me3-type of heterochromatin. This observation is in agreement with the recent findings that only SINE-rich regions of the genome are associated with acetylated H4 histone, a transcribed marker located in euchromatin regions of the genome [14]. Taken together, these findings imply that both L1 and LTR are targeted for epigenetic silencing and that different retrotransposons are packaged as chromatin with different repressive marks. On the basis of these findings we propose a model in which L1 elements are enriched with H2A.Z in the form of H3K9me3 and HP1α-related constitutive heterochromatin, while LTR elements are associated with H2A and the H4K20me3-type of facultative heterochromatin.

## Discussion

The fundamental unit of chromatin is an array of tandem nucleosomes each composed of two copies of the core histones H2A, H2B, H3 and H4 wrapped around genomic DNA. Changes in histone composition and nucleosomal structure are critical in facilitating DNA replication and transcription control. This is mainly governed by PTMs on amino-terminal tails of histones, where they are presumably more accessible to DNA-binding proteins such as transcription factors. The histone modifications like H3K4me2 and H3K9me3 are known to be associated with active and inactive promoters of protein-coding genes, respectively. In addition, the replacement of core histone H2A with the variant histone H2A.Z contributes a specific structure and function to the chromatin [9]. Studies carried out in mouse ES cells have shown that the presence of H2A.Z in the promoter regions of key developmental genes represses their transcription [15]. To reinforce the state of repression, H2A.Z also interacts with facultative polycomb group (PcG) proteins or the constitutive heterochromatin protein HP1α to maintain the condensed structure of chromatin [3,15]. The data presented in this study show that H2A.Z also plays an important role in protecting the genome by repressing the transcriptional activity of mouse L1 elements. Notably, our study reveals that H2A.Z co-localizes with the repressive histone markers H3K9me3 and HP1α. Co-occupancy of both H2A.Z and HP1α suggests that LINE-containing genomic DNA could be involved in the formation of constitutive heterochromatin to keep L1 elements in a silenced state. Support for this notion comes from the finding that, in *Drosophila*, H2A.Z with HP1α are specifically recruited to exogenous transgene arrays and inhibit their expression by assembling the transgenes into ectopic heterochromatin [16].

Cells normally utilize a number of silencing mechanisms to keep different retrotransposons in a transcriptionally silenced state. Our data demonstrate that the core histone H2A co-localizes with heterochromatic H4K20me3 at LTR elements. The presence of H4K20me3 at genomic regions is often considered to be an important component of a silencing pathway that can index pericentric heterochromatin structures [13]. Several studies carried out in *Drosophila* and mouse cells have also shown that H4K20me3 is a key player in genome maintenance by assembling the inactive X chromosome into facultative heterochromatin [13]. In mouse MEF cells, silent promoters of imprinted genes are marked by the presence of H4K20me3, while active promoters lack this repressive modification [17]. We found in this study that H4K20me3 is also enriched at the promoters of both the *Mm*ERV and IAP classes of LTR elements, suggesting that H4K20me3 can contribute to heterochromatin formation of LTR elements, thereby keeping them under the control. At present it is not clear whether the presence of H4K20me3 is a common factor silencing of all other classes of LTR retrotransposons. In addition, it is also unclear why the repressive H4K20me3 marker targets only LTR elements and not L1 elements. One can speculate that the differences in occupancy of histone marks might be influenced by different methylation status of the retrotransposon promoters. Thus, further studies are required to characterize the different classes of retrotransposons in various types of mouse cells. This may also have particular relevance to cancer where different cells express different classes of retrotransposons during cancer progression. Nonetheless, the data presented in our study show for the first time that different epigenetic silencing mechanisms operate in the mouse genome to keep L1 and LTR retrotransposons in a silenced state.

## Abbreviations

ChIP: Chromatin immunoprecipitation; IAP: Intracisternal A-particle; LINE-1: Long interspersed nuclear element-1; LTR: Long terminal repeat; MmERV: Mouse endogenous retrovirus; PTM: Post-translational modification; SeqChIP: Sequential chromatin immunoprecipitation.

## Competing interests

The author declares that he has no competing interests.

## Supplementary Material

Additional file 1: Figure S1 (A) Size distribution of chromatin fragments. Cells were fixed for 10 minutes with 5 mM dimethyl 3,3’-dithiobispropionimidate (DTBP) for a protein-protein cross-linking, followed by DNA-protein cross-linking with 1% formaldehyde. The chromatin samples were sheared for 5, 7, 10, and 12 minutes using the Diagenode’s Biorupter Sonicator to optimize chromatin shearing conditions. The sheared and unsheared chromatin samples were subjected to crosslink reversal and Proteinase K and RNaseA treatments. DNA samples were resolved on a 1.2% agrose gel stained with ethidium bromide to visualize the optimal size distribution of chromatin fractions. The marker is a 1 kb-plus DNA ladder (Invitrogen). DNA sonicated for 10 minutes produced chromatin fragments in a range of 200 to 600 bp and was chosen for all ChIP experiments. (B) The specificity of H2A and H2A.Z antibodies was evaluated by 35 cycles of qPCR with primer set targeting to 166 bp of GADPH promoter. The constitutively transcribed GADPH gene is specifically associated with H2A.Z [18] and thus serves as positive control in the ChIP assay. The sonicated chromatin was immunoprecipitated with mouse IgG, anti-H2A and anti-H2A.Z antibodies. H2A.Z-associated DNA fragments reproducibly generated GADPH promoter DNA, while ChIP performed with H2A or with negative IgG did not. Graphs represent the results of three replicates. Click here for file
