# Supplementary figures and images for: Distinctive patterns of epigenetic marks are associated with promoter regions of mouse LINE-1 and LTR retrotransposons
Source: Mob DNA. 2013 Dec 2;4:27. doi: 10.1186/1759-8753-4-27 (PMC4177394; doi:10.1186/1759-8753-4-27)

Additional Figure 1

A

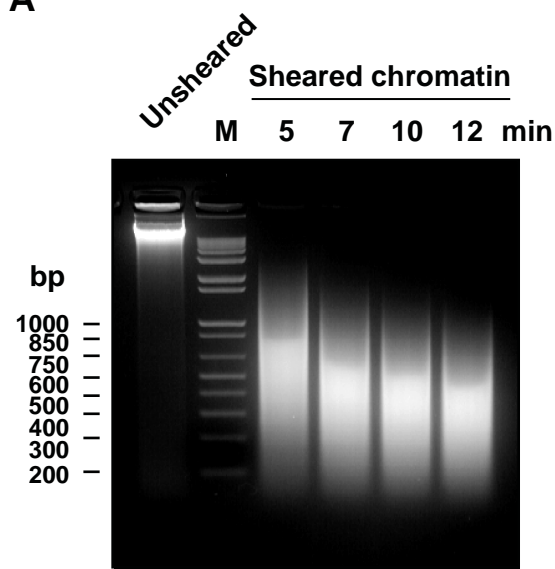

B

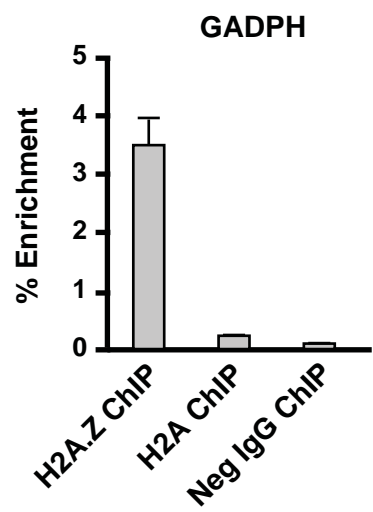

Supplement: Additional file 1: Figure S1 — (A) Size distribution of chromatin fragments. Cells were fixed for 10 minutes with 5 mM dimethyl 3,3’-dithiobispropionimidate (DTBP) for a protein-protein cross-linking, followed by DNA-protein cross-linking with 1% formaldehyde. The chromatin samples were sheared for 5, 7, 10, and 12 minutes using the Diagenode’s Biorupter Sonicator to optimize chromatin shearing conditions. The sheared and unsheared chromatin samples were subjected to crosslink reversal and Proteinase K and RNaseA treatments. DNA samples were resolved on a 1.2% agrose gel stained with ethidium bromide to visualize the optimal size distribution of chromatin fractions. The marker is a 1 kb-plus DNA ladder (Invitrogen). DNA sonicated for 10 minutes produced chromatin fragments in a range of 200 to 600 bp and was chosen for all ChIP experiments. (B) The specificity of H2A and H2A.Z antibodies was evaluated by 35 cycles of qPCR with primer set targeting to 166 bp of GADPH promoter. The constitutively transcribed GADPH gene is specifically associated with H2A.Z [18] and thus serves as positive control in the ChIP assay. The sonicated chromatin was immunoprecipitated with mouse IgG, anti-H2A and anti-H2A.Z antibodies. H2A.Z-associated DNA fragments reproducibly generated GADPH promoter DNA, while ChIP performed with H2A or with negative IgG did not. Graphs represent the results of three replicates. [file 1759-8753-4-27-S1.pdf]
